# Adiposity and Age Explain Most of the Association between Physical Activity and Fitness in Physically Active Men

**DOI:** 10.1371/journal.pone.0013435

**Published:** 2010-10-18

**Authors:** José A. Serrano-Sánchez, Safira Delgado-Guerra, Hugo Olmedillas, Amelia Guadalupe-Grau, Rafael Arteaga-Ortiz, Joaquín Sanchis-Moysi, Cecilia Dorado, José A. L. Calbet

**Affiliations:** 1 Department of Physical Education, University of Las Palmas de Gran Canaria, Las Palmas de Gran Canaria, Spain; 2 Department of Physics, University of Las Palmas de Gran Canaria, Las Palmas de Gran Canaria, Spain; Universidad Europea de Madrid, Spain

## Abstract

**Background:**

To determine if there is an association between physical activity assessed by the short version of the International Physical Activity Questionnaire (IPAQ) and cardiorespiratory and muscular fitness.

**Methodology/Principal Findings:**

One hundred and eighty-two young males (age range: 20–55 years) completed the short form of the IPAQ to assess physical activity. Body composition (dual-energy X-Ray absorptiometry), muscular fitness (static and dynamic muscle force and power, vertical jump height, running speed [30 m sprint], anaerobic capacity [300 m running test]) and cardiorespiratory fitness (estimated VO_2_max: 20 m shuttle run test) were also determined in all subjects.

Activity-related energy expenditure of moderate and vigorous intensity (EEPA_moderate_ and EEPA_vigorous_, respectively) was inversely associated with indices of adiposity (r = −0.21 to −0.37, P<0.05). Cardiorespiratory fitness (VO_2_max) was positively associated with LogEEPA_moderate_ (r = 0.26, P<0.05) and LogEEPA_vigorous_ (r = 0.27). However, no association between VO_2_max with LogEEPA_moderate_, LogEPPA_vigorous_ and LogEEPA_total_ was observed after adjusting for the percentage of body fat. Multiple stepwise regression analysis to predict VO_2_max from LogEEPA_walking_, LogEEPA_moderate_, LogEEPA_vigorous_, LogEEPA_total_, age and percentage of body fat (%fat) showed that the %fat alone explained 62% of the variance in VO_2_max and that the age added another 10%, while the other variables did not add predictive value to the model [VO_2_max  = 129.6−(25.1× Log %fat) − (34.0× Log age); SEE: 4.3 ml.kg^−1^. min^−1^; R^2^ = 0.72 (P<0.05)]. No positive association between muscular fitness-related variables and physical activity was observed, even after adjusting for body fat or body fat and age.

**Conclusions/Significance:**

Adiposity and age are the strongest predictors of VO_2_max in healthy men. The energy expended in moderate and vigorous physical activities is inversely associated with adiposity. Muscular fitness does not appear to be associated with physical activity as assessed by the IPAQ.

## Introduction

Assessment of physical activity (PA) during daily life is crucial to unravel how physical activities may influence present and future health. Several procedures have been used to assess PA which include behavioural observation, questionnaires in the form of diaries, recall questionnaires and interviews, and diverse techniques based on the assessment of motion (accelerometers), heart rate, and energy expenditure (calorimetry and doubly labelled water) [1]. Physical activity questionnaires are the most used, however, their reliability and validity is low [2]–[4]. The validity of most PA questionnaires have been established using techniques to assess the amount of daily energy expenditure such as doubly labeled water [5], [6] and heart rate [7] but also by determining if there is an association between the rating in the questionnaires and the expected effects of an active life style on fitness-related variables such as VO_2_max [8], [9] and body composition [10] as indices of cardiorespiratory fitness, whilst other aspects of fitness like muscle strength and power (muscular fitness) have been largely ignored. An International Consensus Group, which met in Geneva in 1998, developed the International Physical Activity Questionnaire (IPAQ). This questionnaire has a good reliability index [11]–[14] and has been validated against direct measurements of PA by accelerometry [2], [12]–[14] and doubly labelled water [4]. A greater level of PA should translate into better fitness indices [15], [16], including muscle strength and power [17]. So far, no single study has determined which components of fitness are associated with the level of PA assessed with the short version of the IPAQ. This information is important to define what aspects of fitness appear to be influenced by daily PA as determined by general questionnaires, such as the IPAQ. Lack of association between component/s of fitness with PA assessed with the short version of the IPAQ could indicate that either these components are not influenced by physical activity or that the IPAQ is not sensitive enough to detect the influence of PA on some components of physical fitness.

Therefore, the main objective of the present investigation was to determine if there is an association between PA assessed by the short version of IPAQ cardiorespiratory and muscular fitness. Another aim was to determine if the potential associations between PA and fitness are explainable by differences in the level of adiposity. Since reduced PA is associated with accumulation of fat mass and fat mass has a negative influence in fitness tests, we hypothesised that adiposity could explain part or most of the associations observed between PA and fitness in healthy men.

## Materials and Methods

### Subjects

One hundred eighty-two Caucasians males aged 31±7.2 years (mean ± SD; range: 20–55 years) from the island of Gran Canaria agreed to participate in the study. Their cardiorespiratory fitness was similar to that reported for the male population of the United States (estimated mean VO_2_max of 42 ml.kg^−1^.min^−1^, for a mean age of 33 years) according to the National Health and Nutrition Examination Survey (NHANES) 1999–2002 [18]. They were recruited between physically active university students, sports clubs and police officers in Gran Canaria (Spain). The health status of each participant was established by a medical history and physical examination. Subjects taking any kind of medications or having any chronic disease or hypertension were excluded. The study was approved by the ethical committee of the University of Las Palmas de Gran Canaria. All volunteers provided their written informed consent before participation in the study.

### Ethics Statement

The study was performed in accordance with the Helsinki Declaration of 1975, last modified in 2000, as regards the conduct of clinical research, being approved by the Ethical Committee of the University of Las Palmas de Gran Canaria. All volunteers provided their written consent before participation in the study.

### Data Collection

Each subject visited at least in two occasions our laboratory. During the first visit, the short version of the IPAQ was completed, and then a complete anthropometric and body composition assessment was performed. Thereafter, their maximal dynamical strength, jump performance, and 300 m running time were determined. The second visit was used to assess their maximal running speed and maximal aerobic power.

### Questionnaire: Scoring and Data Reduction

Overall PA was assessed using the short last seven days self-administered format of the International Physical Activity Questionnaire (IPAQ) (Spanish version) [19]. The short version (4 items) provides information on the time spent walking, in vigorous- and moderate- intensity activity and in sedentary activity (time spent sitting per week). Note that the sitting question was developed as separate indicator and not as part of the summed PA score. The data collected were used to estimate total weekly PA by weighting the reported minutes per week within each activity category. Metabolic equivalent (MET) levels were obtained from the document “Guidelines for the data processing and analysis of the International Physical Activity Questionnaire” available online at http://www.ipaq.ki.se. The weighted MET-minutes per week (MET·min·wk^−1^) was calculated as duration x frequency per week x MET intensity, which were summed across activities to produce a weighted estimate of total PA from all reported activities per week (MET·min·wk^−1^). IPAQ defines three levels of PA: Low, Moderate or High. The IPAQ Research committee propose two criteria for classification as high: a) vigorous-intensity activity on at least 3 days achieving a minimum total PA of at least 1500 MET-minutes/week or b) 7 or more days of any combination of walking, moderate-intensity, or vigorous-intensity activities achieving a minimum total PA of at least 3000 MET-minutes/week. Moderate category is defined as doing some activity, more than the low category. The pattern of activity to be classified as moderate is either of the following criteria: a) 3 or more days of vigorous-intensity activity of at least 20 minutes per day or b) 5 or more days of moderate-intensity activity and/or walking of at least 30 minutes per day or c) 5 or more days of any combination of walking, moderate-intensity or vigorous intensity activities achieving a minimum total PA of at least 600 MET-minutes/week (www.ipaq.ki.se). The low PA category includes the subjects not achieving the minimum for the moderate PA category.

### Physical Fitness

#### Anaerobic capacity

A three hundred meter running test was used to estimate the anaerobic capacity [20], because the anaerobic capacity is the first determinant of performance in maximal all-out efforts eliciting exhaustion between 30 and 60 seconds [21]. The test was performed on a 400 m track, and timings were measured manually. Subjects were asked to run the 300 m as fast as possible. The intraclass correlation coefficient alpha (Cronbach) was 0.94 in 20 physical education students who repeated the test two times in different days.

#### Running speed test

The time needed to cover 30 meters (T_30_) was measured with photoelectric cells (General ASDE, Valencia). The timer was automatically activated when the subject crossed the first cell, and every 5 meters thereafter. Subjects were motivated to run as fast as they could, and the best performance achieved in three trials, separated by at least 1 min rest period, was taken as the representative value of this test. The intraclass correlation coefficient alpha (Cronbach) for the running times at 5, 10, 15, 20, 25 and 30 m were: 0.91, 0.97, 0.98, 0.99, 0.99 and 0.99, as determined previously in 14 physical education students who repeated the test three times [22].

#### Aerobic maximal power

The maximal oxygen uptake (VO_2_max) was estimated using a maximal multistage 20-m shuttle run test [23]. Subjects were asked to run back and forth on a 20 m course and be on the 20 m line coinciding with beeps emitted from a tape. The frequency of the sound signals increased in such a way that running speed started at 8.5 Km·h^−1^ and was increased by 0.5 Km·h^−1^ every minute. The length of time the subjects were able to run for was recorded to calculate the VO_2_max, using the equation VO_2_max (ml.kg^−1^.min^−1^) from the speed (S, km.h^−1^) corresponding to the final stage (VO_2_max = 31.025+6.003S−58.464) [23]. The maximal multistage 20-m shuttle run test has been shown to be valid and reliable for the estimation of the VO_2_max with a reliability coefficient of 0.95 [23].

#### Isometric force and squat and counter movement jumps

The force generated during maximal isometric force (MIF), and the height of jumping during vertical Squat Jumps (SJ) and Counter Movement Jumps (CMJ), were measured with a force plate (Kistler 2822A1-1, Winterthur, Switzerland) and sampled at 500 Hz. The MIF starts in the squat position with the knees bent at 90°. SJ and CMJ jumps were chosen to assess muscle power due to their high reliability [24]. The SJ starts from the squat position with the knees bent at 90°. The CMJ starts from a standing position allowing for counter movement, with the intention of reaching knee bending angles of around 90° prior to impulsion. The best of three attempts in force and jump tests were selected for further analysis. The jumping heights (Hj) generated were determined by integration of the vertical ground reaction forces in the best of three trials in both kinds of jumps, SJ and CMJ [25]. During the push-off phase, the vertical velocity of the centre of masses was determined by integration over time of the acceleration, which, in turn, was calculated from the ground reaction force signal. Instantaneous jump power was continuously calculated as the product of vertical ground reaction force and center of mass velocity [26]. The intraclass correlation coefficients alpha (Cronbach) for the MIF and the height jumped during the vertical jumps were all above 0.98, when measured three times in 10 physical education students.

### Body Composition

#### Percentage of trunk and whole body fat

The percentage trunk and whole body fat were measured using dual-energy X-ray absorptiometry (DXA) (QDR-1500, Hologic Corp., Software version 7.10, Waltham, MA) as described elsewhere [27], [28]. The DXA scanner was calibrated using a lumbar spine phantom. Subjects were scanned in supine position and the scans were performed in high resolution. Fat mass (%) were calculated from total and regional analysis of the whole body scan [27].

#### Anthropometric measurements

Height, weight, Body Mass Index (BMI), thorax, waist, and hip circumferences were measured by the Advanced O-Scale Physique Assessment System. Waist to Hip ratio was calculated using the formula W-H =  waist/hip (cm).

### Statistical Analysis

Mean and standard deviation (SD) are given as descriptive statistics. All variables were checked for normal distribution by the Shapiro-Wilks test. Due to non-normal distribution, age, waist circumference, hip circumference, 30 and 300 m running times, EEPA_walking_, EEPA_moderate_, EEPA_vigorous_ and EEPA_total_, and the percentage of whole body and trunk fat mass were logarithmically transformed. The association between the estimated energy expenditure EEPA_walking_, EEPA_moderate_, EEPA_vigorous_ and EEPA_total_ with body composition and fitness variables was determined with the Pearson's correlation test. Differences in body composition and fitness variables between the three IPAQ (low, moderate and high) PA categories were tested with analysis of variance with the Bonferroni pot hoc test. In addition, ANCOVA with age and the percentage of body fat as covariables was also carried out to determine if differences in fitness variables between the three IPAQ physical activity categories could be explained by the influence of these two potential confounders. Stepwise multiple regression analysis was used to determine which fitness and body composition variables had greater predictive value for VO_2_max. Significant differences were assumed when p<0.05. The statistical power for correlation analyses was above 0.8 (for r≥0.20) in all analyses, except for the EEPA_Vigorous_. The statistical power for correlation analyses involving the EEPA_Vigorous_ group was >0.8 only for r>0.32. Data were analyzed with SPSS version 15.0.

## Results

### Anthropometrical Variables and Body Composition

Pearson's correlation coefficients between physical and anthropometric characteristics and estimated energy expenditure by IPAQ are shown in Table 1. There was an inverse association between indicators of adiposity (BMI, waist and hip circumferences, waist-to-hip ratio, and percentage of fat in the trunk and whole body) and LogEEPA_moderate_. Similar associations were observed between the waist-to-hip ratio, and the percentage of fat in the trunk and whole body and LogEEPA_vigorous_. The strength of these associations was not modified when the energy expended in moderate and vigorous physical activities was added. No association between LogEEPA_total_ and anthropometrical and body composition variables was observed. The percentage of fat mass in trunk and whole body was positively associated with LogEEPA_walking_.

**Table 1:** Pearson's correlation coefficient matrix between anthropometric characteristics, body composition and estimated METs expended per week derived from the IPAQ by activity type or intensity.

Variables	EEPA_Walking_ h	EEPA_Moderate_ h	EEPA_Vigorous_ h	EEPA_Total_ h
Number of subjects	147	146	73	176
Age (year) h	0.39 a	−0.11	0.01	0.23 a
Weight (kg)	0.15	−0.16	−0.06	0.03
BMI (weight(kg)/height(m^2^))	0.13	−0.23 a	−0.16	−0.05
Thorax circumference (cm)	0.10	−0.15	0.10	0.02
Waist circumference (cm) h	0.21 a	−0.27 a	−0.22	−0.07
Hip circumference (cm) h	0.06	−0.21 a	−0.12	−0.08
Waist to Hip ratio	0.28 a	−0.21 a	−0.26 a	−0.03
TF (%) h	0.28 a	−0.29 a	−0.29 a	−0.09
BF (%) h	0.26 a	−0.28 a	−0.37 a	−0.06

BMI, body mass index; TF (%), percentage of trunk fat; BF (%), percentage of body fat.

a p≤0.05;

h Logarithmic transformation; EEPA, energy expenditure in each physical activity type (MET·min·wk^−1^).

### Cardiorespiratory Fitness

Pearson's correlation coefficients between physical fitness variables and estimated energy expenditure by IPAQ are shown in Table 2. Cardiorespiratory fitness was positively associated with LogEEPA_moderate_ (r = 0.26, P<0.05) and LogEEPA_vigorous_ (r = 0.27, P<0.05). This association was maintained with EEPA vigorous after adjusting for age (r = −0.24, P<0.05). However, no association between VO_2_max and LogEEPA_moderate_ and vigorous was observed after adjusting for the percentage of body fat. There was a negative association between VO_2_max and LogEEPA_walking_ (r = −0.31, P<0.05), even after adjusting for the percentage of body fat (r = −0.40, p<0.05).

**Table 2:** Pearson's correlation coefficient matrix between physical fitness variables and estimated METs expended per week derived from the IPAQ by activity type or intensity.

Variables	EEPA_Walking_ h	EEPA_Moderate_ h	EEPA_Vigorous_ h	EEPA_Total_ h
**Unadjusted**				
MIF (KgF)	−0.12	−0.01	0.05	−0.09
HSJ (m)	−0.08	0.10	0.12	0.03
HCMJ (m)	−0.15	0.08	0.02	−0.09
V_30_ (s) h	0.22 a	−0.04	0.11	0.13
V_300_ (s) h	0.19 a	−0.15	−0.18	−0.05
VO_2_max (ml·kg^−1^·min^−1^)	−0.31 a	0.26 a	0.27 a	0.07
**Age adjusted**				
MIF (KgF)	−0.26	0.15	0.04	−0.06
HSJ (m)	0.06	0.15	0.17	0.14
HCMJ (m)	0.13	−0.06	0.00	−0.03
V_30_ (s) h	−0.09	0.11	0.16	−0.01
V_300_ (s) h	0.16	−0.12	−0.24	−0.24 a
VO_2_max (ml·kg^−1^·min^−1^)	−0.25	0.22	0.31 a	0.25 a
**% Fat adjusted**				
MIF (KgF)	−0.24	0.18	0.08	−0.09
HSJ (m)	−0.03	0.15	0.05	−0.01
HCMJ (m)	−0.07	0.02	−0.12	−0.13
V_30_ (s) h	0.19	−0.05	0.26	0.19 a
V_300_ (s) h	0.24	−0.03	0.01	0.00
VO_2_max (ml·kg^−1^·min^−1^)	−0.40 a	0.07	−0.14	−0.00
**Age plus % Fat adjusted**				
MIF (KgF)	−0.27	0.18	0.08	−0.05
HSJ (m)	0.08	0.11	0.12	0.08
HCMJ (m)	0.13	−0.07	−0.01	−0.01
V_30_ (s) h	−0.08	0.07	0.13	0.05
V_300_ (s) h	0.12	0.03	−0.08	−0.15 a
VO_2_max (ml·kg^−1^·min^−1^)	−0.24	−0.04	0.01	−0.14

MIF, maximal isometric force; HSJ, height in squat jump; HCMJ, height in counter movement jump; V_30_, velocity in 30 m running speed test; V_300_, velocity in 300 m running test; VO_2_max, maximum oxygen uptake.

a *p*≤0.05;

h Logarithmic transfor-mation; EEPA, energy expenditure in each physical activity type (MET·min·wk^−1^).

**Figure 1:**
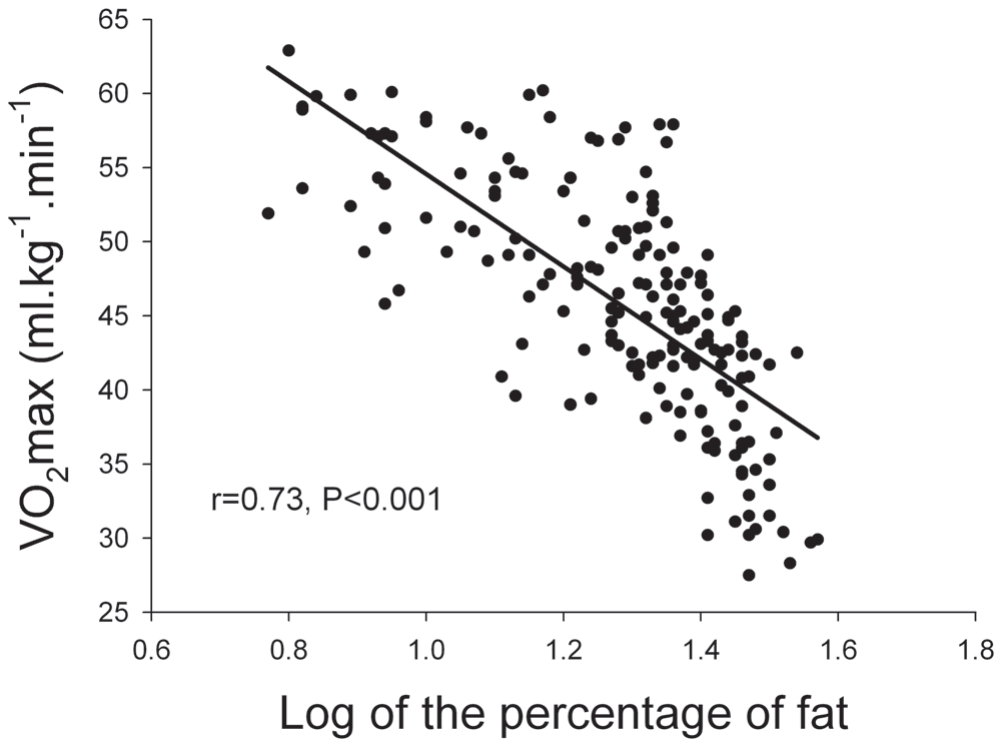
Relationship between VO_2_max and the logarithm of the percentage of body fat (unadjusted values).



Maximal oxygen upytake (ml.kg^−1^.min^−1^) could be predicted from EEPA_walking_ and EEPA_vigorous_ as shown by stepwise multiple regression analysis (VO_2_max = 51.5−(0.79× LogEEPA_walking_) + (6.5× LogEEPA_vigorous_, R^2^ = 0.34, P<0.01), whilst EEPA_moderate_ was excluded from the regression model. However, there was also a strong inverse association between VO_2_max and the logarithm of the percentage of body fat (r = −0.73, P<0.05) (Fig. 1), which was reduced to r = −0.63 (P<0.05) after adjusting for the logarithm of age, or for both the logarithm of age and the log of (EEPA_moderate_ + EEPA_vigorous_) (r = −0.63, P<0.05). Thus, relative VO_2_max (ml.kg of body mass^−1^. min^−1^) is associated more strongly to the percentage of body fat than to weekly energy expended in moderate and vigorous physical activities in physically active men. Stepwise multiple regression analysis to predict VO_2_max from LogEEPA_walking_, LogEEPA_moderate_, LogEEPA_vigorous_, LogEEPA_total_, age and percentage of body fat (%fat), indicated that the %fat explained 62% of the variance in VO_2_max and the age another 10%, while the other variables did not improve the predictive value of the model.

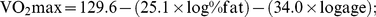



R^2^ = 0.72 (P<0.05).

SEE (standard error of estimate): 4.3 ml.kg^−1^.min^−1^.

Only LogEEPA_vigorous_ was significantly associated to the percentage of body fat after adjusting for VO_2_max (r = −0.26, P<0.05).

### Muscular Fitness

No positive association between muscular fitness-related variables (maximal isometric force, vertical jump performance, 30 m and 300 m running speed) and PA was observed, even after adjusting for body fat (Table 2). Only the 300 m running speed was associated with EEPA_total_ after adjusting for body fat and age (r = 0.15, P<0.05). The percentage of body fat was negatively associated to LogEEPA_moderate_ (r = −024, p<0.01). LogEEPA_vigorous_ was significantly associated to the percentage of body fat after adjusting for the 300 m running performance (r = −0.33, p<0.01).

### Differences in Physical Fitness Between the Three IPAQ Physical Activity Categories

From the IPAQ outcomes, 43 subjects were classified into the Low (age = 30±7.5 yr; mean ± SD), 91 into the Moderate (age = 30±7.2 yr; mean ± SD), and 48 into the High (age = 33±6.0 yr; mean ± SD) PA levels. The subject's age, anthropometric, physical fitness and body composition data by PA category are summarized in Table 3. The moderately physically active group had higher VO_2_max than the low physically active group and run the 30 m test faster than the highly active group. Waist and Hip circumferences, as well as the percentage of whole body and trunk fat mass were greater in the low compared to the moderately active group. No differences between the three PA categories were observed in weight, BMI, waist to hip ratio, maximal isometric force, jumping height in squat and counter movement jump, and 300 m running times. However, the subjects from the high PA category were three years older than the subjects from the low and moderate PA category. Adjusting for age as covariate did not change the results with the exception of age-adjusted VO_2_max, age-adjusted percentage of body fat and age-adjusted percentage of trunk fat mass, which were significantly lower in the low compared to both the moderate and high PA groups.

**Table 3:** Subjects' physical characteristics and indicators of physical fitness by IPAQ classified physical activity categories (mean ± SD).

Variables	Low			Moderate			High			N
Age (year)	30.34^a^	±	7.53	30.25 a	±	7.22	33.62	±	6.04	43-91-48
Weight (kg)	83.02	±	10.58	78.68	±	10.17	80.57	±	9.97	43-91-48
Height (cm)	177.2	±	0.8	176.4	±	0.6	177.5	±	0.8	43-91-48
BMI (weight(kg)/height(m^2^))	26.46	±	3.41	25.31	±	3.33	25.55	±	2.69	43-91-48
Thorax circumference (cm)	101.94	±	8.89	98.87	±	7.44	101.77	±	6.64	43-91-48
Waist circumference (cm)	92.33 b	±	8.65	87.75	±	9.06	89.32	±	7.84	43-91-48
Hip circumference (cm)	101.01 b	±	8.22	97.86	±	5.76	97.91	±	5.48	43-91-48
Waist to Hip ratio (cm)	0.91	±	0.06	0.90	±	0.05	0.91	±	0.05	43-91-48
MIF (Kgf)	112.11	±	16.49	104.13	±	24.39	103.98	±	20.79	43-91-48
HSJ (m)	0.28	±	0.05	0.29	±	0.05	0.28	±	0.06	43-91-48
HCMJ (m)	0.32	±	0.06	0.33	±	0.06	0.31	±	0.07	43-91-48
V_30_ (seg)	4.56	±	0.28	4.53 a	±	0.29	4.66	±	0.36	43-91-48
V_300_ (seg)	51.68	±	7.04	50.04	±	6.68	51.74	±	10.07	43-91-48
VO_2_max (ml·kg^−1^·min^−1^)	43.17 b	±	7.68	47.43	±	8.00	45.58	±	7.45	43-91-48
TF (%)	23.44 b	±	9.12	18.46	±	9.41	20.73	±	9.50	43-91-48
BF (%)	23.11 b	±	6.90	19.30	±	7.09	20.36	±	7.41	43-91-48

BMI, body mass index; MIF, maximal isometric force; HSJ, height in squat jump; HCMJ, high in counter movement jump; V_30_, velocity in 30 m running speed test; V_300_, velocity in 300 m running test; VO_2_max, maximum oxygen uptake; TF (%), percentage of trunk fat; BF (%), percentage of body fat.

a *p*≤0.05 compared to High;

b *p*≤0.05 compared to Moderate.

When adjusted only for the percentage of body fat, running times in 30 m were lower in the low than in the high PA group. However, after adjusting for both age and percentage of body fat no between-groups differences were observed in 30 m running times. Adjusting for age or the percentage of body fat or both (age and percentage of body fat) did not change the results regarding maximal isometric force and jumping height.

## Discussion

There are three main findings in this study. First, the energy expended in moderate and vigorous physical activities as estimated by the IPAQ is inversely associated with adiposity in physically active men. Second, that adiposity explains most of the association between PA and fitness in physically active men. And third, that there is no association between muscular fitness-related variables and energy expenditure in any of the three categories distinguished by the short version of the IPAQ.

In partial agreement with our results, Papathanasiou et al. [29] reported significant correlations between the total and vigorous physical activities (Greek version of IPAQ-short) and maximal treadmill time (assessed with Bruce's treadmill test) (0.35 to 0.43) in young health-science students (20–29 years old). Moderate and low physical activities correlations were poor and non-significant [29]. Also in young adults, essentially similar results were reported by Kurtze et al [13]. However, it has been also reported lack of correlation between the percentage of body fat and IPAQ variables from the long [30] and short versions of the IPAQ [31].

Our study concurs with the recent article by Nokes [32] who observed that the percentage of body fat accounted for 47% of the variability in cardiorespiratory fitness in 247 non-obese women (BMI<30; mean age of 40±3 years [±SD]), in which cardiorespiratory fitness was assessed by measuring VO_2_max with an incremental exercise test to exhaustion and the percentage of body fat with air-displacement plethysmography. In this study daily PA was measured by accelerometry during 7 days. These authors observed that the strength of association between PA and cardiorespiratory fitness was reduced from r^2^ = 0.11 to r^2^ = 0.056, when adjusted for the percentage of body fat. In the present investigation, similar results have been obtained with a population composed only by physically active men with a wide range of PA and fitness levels.

Our study also agrees with the work of Nokes [32] in highlighting the importance that the intensity of the exercise has for association between cardiorespiratory fitness and PA. In fact, in our study only the weekly energy expenditure (as estimated by the IPAQ) in moderate and vigorous physical activities was positively associated with VO_2_max. After adjustment for VO_2_max only the energy expenditure in vigorous PA was associated to a lower percentage of body fat. Some interventional studies have shown that high intensity intermittent exercise can be even more effective that than steady state exercise in eliciting a reduction of fat mass, both types of exercise requiring a similar energy expenditure [33] or even with lower energy expenditure in the high intensity intermittent exercise [34]. This intensity-effect may be related to greater energy deficit incurred with high intensity exercise programs. It seems that the exercise-related increase in caloric intake does not compensate for the increased energy expenditure of the high intensity exercise programs due to changes in appetite [35], [36].

We expected highly active subjects to achieve higher levels of performance in tests measuring diverse aspects of physical fitness including muscular strength and power, endurance (aerobic power, i.e. VO_2_max), anaerobic capacity and sprinting capacity (running speed in 30 m). In addition, we also presumed that the most active subjects according to IPAQ would also have a lower percentage of body fat, and vice versa. In agreement, the level of performance achieved in VO_2_max (cardiorespiratory fitness) by the distinct IPAQ categories was significantly different, with the subjects assigned to the low PA levels by IPAQ showing the lowest mean values of cardiorespiratory fitness. However, the mean difference in VO_2_max between the IPAQ physical activity categories were rather small and after accounting for the percentage of body fat as covariable no differences in cardiorespiratory and muscular fitness were observed between the three IPAQ physical activity categories. This could indicate that one important mechanism by which PA contributes to improve cardiorespiratory fitness is by reducing fat mass. This interpretation is further supported by the regression analysis showing that both the percentage of body fat and age were able to explain 72% of the variance in VO_2_max in our population, whilst PA did not contribute any additional predictive value. The confounding effect that adiposity may have on physical fitness is further emphasized by some studies showing that obese subjects compared to non-obese controls have reduced VO_2_max when expressed per kg of body mass but similar VO_2_max when expressed per kg of lean mass [37]. Likewise, adiposity explains great part of the difference in physical fitness between physically active and sedentary children [38].

Although good correspondence between the time expended in moderate activities and accelerometry measurements have been reported for the short IPAQ [14], we have observed in several instances that the subjects classified by IPAQ into the moderate PA category performed better in the physical fitness tests than the subjects classified by IPAQ into the highly active category. Similar problems have been reported using the long form of IPAQ to assess PA in patients with chronic fatigue syndrome [39] and with short form of the IPAQ in healthy Swedish adults [40]. This may be due to the intrinsic difficulty that entails the assessment of PA by questionnaire [3]. Several studies report that the IPAQ significantly overestimated self-reported time spent in physical activities [40]–[42] while inactivity is highly underreported [14]. This is likely due to the fact that it is difficult to obtain a good assessment of low and moderate PA using self-administered questionnaires [10], because these activities are being accumulated throughout the day and the number and diversity of these activities is enormous, resulting in a poor recall. In fact, it has been shown that activity-related energy expenditure measured with the long versions of the IPAQ underestimates the actual energy expenditure measured with doubly labelled water by 27% [4]. This error is lower in subjects with low levels of PA, but greater in those with high levels of PA [4].

As a novelty, this study reports no statistically significant differences in the 30 m running test between the IPAQ PA categories were observed after adjusting for differences in both the percentage of whole body fat and age. Another novel finding is that the 300 m running speed was associated with EEPA_total_ after adjusting for body fat and age. No between-categories significant differences were observed in the rest of the muscular fitness-related variables (maximal isometric force and jumping height) even after accounting for age, percentage of body fat or both (age and percentage of body fat) as covariates. The latter highlights the difficulty that entails the assessment of high intensity physical activities with physical activity questionnaires.

There was a negative association between VO_2_max and LogEEPAwalking even after adjusting for the percentage of body fat. Walking exercise alone is unlikely to enhance VO_2_max [43]–[45] but walking should not have negative effects on VO_2_max. Thus the negative association in this study could indicate that subjects with low cardiorespiratory fitness chose to walk rather than to engage in moderate or vigorous intensity PA.

This study has several limitations. First, the population studied is not representative of the Spanish population implying that our results have limited generalizability. Second, fitness variables and PA were measured with methods having different levels of reliability and validity. VO_2_max was estimated and not directly measured. For this purpose we used an incremental test to exhaustion which have been shown to be valid and reliable to estimate VO_2_max [23], [46]. Body composition was also measured with a method (dual-energy x-ray absorptiometry) of high validity and reliability (see [47] for references). On the other hand, PA was assessed by questionnaire a procedure with lower reliability and validity than the procedures applied to determine body composition and fitness, limiting the capacity to find associations between PA and fitness variables. Despite these limitations, the percentage of body fat and age explained 72% of variance in VO_2_max, leaving only 28% to be explained by other factors such as PA. PA measured with accelerometry (daily step counts) has been reported to be associated to VO_2_max in Japanese women after adjustment for age (r = 0.55 for, n = 48) [48]. This result can be compared with the association observed in the present investigation between VO_2_max and the daily energy expenditure as assessed by the IPAQ (r = 0.25, age adjusted). However, Cao et al. did not analyze the influence of adiposity in the association between VO_2_max and accelerometry-measured PA [48] and their population was more uniform in terms of BMI than our subjects.

### Conclusions

Adiposity and age are the strongest predictors for VO_2_max in physically active healthy men. Adiposity explains most of the associations between physical activity and fitness in this population. Our results also indicate that the IPAQ physical activity categories correspond relatively well with the level of cardiorespiratory fitness. Muscular fitness does not appear to be associated with physical activity as assessed by the IPAQ.
